# When healthcare providers are supportive, ‘I'd rather not test alone’: Exploring uptake and acceptability of HIV self‐testing for youth in Zimbabwe – A mixed method study

**DOI:** 10.1002/jia2.25815

**Published:** 2021-09-27

**Authors:** Constancia V. Mavodza, Constance R. S. Mackworth‐Young, Tsitsi Bandason, Ethel Dauya, Chido Dziva Chikwari, Mandikudza Tembo, Tsitsi Apollo, Getrude Ncube, Katharina Kranzer, Rashida Abbas Ferrand, Sarah Bernays

**Affiliations:** ^1^ Biomedical Research and Training Institute Harare Zimbabwe; ^2^ Department of Public Health, Environments and Society, Faculty of Public Health and Policy London School of Hygiene and Tropical Medicine London UK; ^3^ Department of Global Health and Development, Faculty of Public Health and Policy London School of Hygiene and Tropical Medicine London UK; ^4^ Clinical Research Department, Faculty of Infectious and Tropical Diseases London School of Hygiene and Tropical Medicine London UK; ^5^ MRC London School of Hygiene and Tropical Medicine London UK; ^6^ HIV and TB Department Ministry of Health and Child Care Harare Zimbabwe; ^7^ Division of Infectious and Tropical Medicine Medical Centre of the University of Munich Munich Germany; ^8^ School of Public Health University of Sydney Sydney New South Wales Australia

**Keywords:** decision‐making, HIV self‐testing, preferences, sequential explanatory mixed method design, youth, Zimbabwe

## Abstract

**Introduction:**

In sub‐Saharan Africa, less than half of young people know their HIV status. HIV self‐testing (HIVST) is a testing strategy with the potential to offer privacy and autonomy. We aimed to understand the uptake and acceptability of different HIV testing options for youth in Harare, Zimbabwe.

**Methods:**

This study was nested within a cluster randomized trial of a youth‐friendly community‐based integrated HIV and sexual and reproductive health intervention for youth aged 16–24 years. Three HIV testing options were offered: (1) provider‐delivered testing; (2) HIVST on site in a private booth without a provider present; and (3) provision of a test kit to test off site. Descriptive statistics and proportions were used to investigate the uptake of HIV testing in a client sample. A focus group discussion (FGD) with intervention providers alongside in‐depth interviews, paired interviews and FGDs with a selected sample of youth clients explored uptake and acceptability of the different HIV testing strategies. Thematic analysis was used to analyse the qualitative data.

**Results:**

Between April and June 2019, 951 eligible clients were tested for HIV: 898 (94.4%) chose option 1, 30 (3.25%) chose option 2 and 23 (2.4%) chose option 3. Option 1 clients cited their trust in the service and a desire for immediate counselling, support and guidance from trusted providers as the reasons for their choice. Young people were not confident in their expertise to conduct HIVST. Concerns about limited privacy, confidentiality and lack of support in the event of an HIV‐positive result were barriers for off‐site HIVST.

**Conclusions:**

In the context of supportive, trusted and youth‐friendly providers, youth clients overwhelmingly preferred provider‐delivered HIV testing over client‐initiated HIVST or HIVST off site. This highlights the importance of listening to youth to improve engagement in testing. While young people want autonomy in choosing when, where and how to test, they do not want to necessarily test on their own. They desire quality in‐person counselling, guidance and support, alongside privacy and confidentiality. To increase the appeal of HIVST for youth, greater provision of access to private spaces is required, and accessible pre‐ and post‐test counselling and support may improve uptake.

## 1 INTRODUCTION

Despite considerable investments to increase HIV testing rates, knowledge of HIV status remains low in sub‐Saharan Africa (SSA) particularly among adolescents and young adults (10–24 years) [1, 2, 3]. In SSA, it is estimated that less than half of youth aged 15–24 years know their HIV status [4]. Barriers to testing in facilities include long waiting times, concerns around privacy and confidentiality, and fear of negative and judgemental interactions with healthcare workers [5, 6, 7].

HIV self‐testing (HIVST), defined as a process where an individual performs an HIV test themselves and interprets the result in private or in the presence of someone they trust (who is not a health provider), has stimulated considerable interest because of its potential to improve uptake of testing [8]. HIVST can use oral fluid or blood‐samples and can overcome existing barriers to youth HIV testing, including through facilitating autonomy and reducing anticipated stigma [9, 10]. It may be an effective way to reach groups commonly described as ‘hard to reach’ [11]. Studies have shown high levels of uptake of HIVST among groups with increased HIV exposure, such as men who have sex with men (MSM), sex workers, adolescents (16–19 years) and pregnant women in SSA [6, 12, 13].

The World Health Organization has recommended that HIVST be integrated as an option in HIV testing services [14]. However, the evidence about HIVST among youth from SSA is limited [12, 15]. Little is known about the relative acceptability and uptake of HIVST and in what context the delivery of HIVST could increase testing uptake specifically among youth. We investigated the uptake and acceptability of provider‐delivered testing compared to HIVST offered as part of an integrated package of health services to youth in community‐based settings in Zimbabwe.

## 2 METHODS

### 2.1 Study setting

This study was embedded within the CHIEDZA (Community based interventions to improve HIV outcomes in youth: a cluster randomised trial in Zimbabwe) trial. The trial aims to investigate the impact of providing a package of HIV and sexual and reproductive health services (SRHs) delivered to youth (16–24 years) in a community‐based setting, on population‐level HIV viral load. The package includes HIV testing and counselling, linkage to care for those who test HIV positive, provision of antiretroviral treatment and adherence support, as well as condoms, family planning, menstrual health products, general health counselling and management of sexually transmitted infections. The trial is being implemented in three provinces in Zimbabwe (Harare, Bulawayo and Mashonaland East). There are a total of 24 clusters (a geographically demarcated area that includes a primary care clinic and a community hall from which services are delivered), which are randomized 1:1 to the intervention package or standard of care (existing health services largely provided by primary healthcare clinics). Each cluster has between 2000 and 4000 youth, representing about 30% of the cluster population. This paper presents the analysis of data collected from all four intervention clusters implementing HIVST within the Harare province from 1st April to 27th June 2019.

Youth aged 16–24 years resident within the cluster boundaries were eligible for CHIEDZA services. Clients who did not know their status and/or had not been tested in the past 6 months were eligible for HIV testing. CHIEDZA's approach is to consistently offer testing to youth and create a safe environment where clients can choose when to take up testing. Three options for HIV testing were offered: (1) provider testing (trained providers performing the test); (2) HIVST on site in a private booth without a provider present; and (3) provision of a test kit to test off site. For all options, an oral mucosal test (OMT) was used and clients were counselled that a reactive test would require confirmation by a blood‐based rapid antibody test as per national guidelines.

Clients who opted for HIVST were given an OMT kit with a unique kit number (recorded by the provider on the client data form). These clients needed to have a sufficiently sophisticated smartphone to access a custom‐built mobile application (ITHAKA) that supported clients to perform HIVST. The ITHAKA application provided pre‐test counselling and instructional videos on the testing process in the local language and guided individuals through the test procedure. Clients who opted to self‐test for HIV on site accessed ITHAKA from a SAMSUNG Galaxy electronic tablet A10 in a private booth. Clients who chose to test off site were required to download the app to their smartphone device, and used an exclusive data voucher to access the ITHAKA application.

### 2.2 Study design

The purpose of the study was to examine young people's preferences in HIV testing method and to understand their reasoning. This was a mixed methods study, where we used an explanatory sequential design to integrate our analysis of quantitative and qualitative data [16, 17]. Quantitative analysis of the routine intervention data showed very low uptake of HIVST. Qualitative methods were used to explain the trends identified in the quantitative data. The study was designed to inform, where feasible, any rapid adjustments that could be made to the delivery of HIVST to improve uptake.

### 2.3 Data collection and analysis

#### 2.3.1 Quantitative data

The primary quantitative outcome of this study was uptake of the three available modes of HIV testing. At each visit, a fingerprint was taken which was automatically converted into a unique client identifier using SIMPRINTS software (Cambridge, UK) to track client service usage across multiple visits. Data were analysed using STATA v14.0 (StataCorp, College Station, TX, USA), and uptake of testing by age, sex and study cluster was computed.

#### 2.3.2 Qualitative data

We used qualitative methods to explore the perceptions and experiences of CHIEDZA providers and clients of the three HIV testing methods. Seven (four females and three males) CHIEDZA health providers participated in a focus group discussion (FGD) to better understand their experiences of providing HIV testing services for youth. Eligible clients could choose to participate in either an FGD or in‐depth interview (IDI). FGDs explored clients’ perceptions of the testing options. Both paired (two participants in one interview) and individual interviews were used to understand individual testing experiences. Four client FDGs were conducted: two with exclusively female clients (*n* = 4, *n* = 7), one with exclusively male clients (*n* = 4) and one mixed FDG (five female clients and one male client). We also conducted two female same‐sex paired IDIs (*n* = 4) and six individual IDIs (*n* = 6). One interview participant was accompanied by her HIV‐positive sister. This is treated as an individual interview as the sister, who had not tested at CHIEDZA, was not interviewed.

When recruitment began (12 June 2019), all youth who had attended CHIEDZA and taken an HIV test at CHIEDZA within the previous 11 weeks were deemed eligible to participate (*n* = 1415). Thirty‐five of these eligible clients were conveniently selected to include variations in age and gender. They were invited to participate by three trained youth researchers aged 18–23 years and all 35 invited initially agreed. Four of them did not attend the interview or said that they no longer had time. Of the 31 who participated, 26/31 were females and 27/31 were 16–19 years old.

The majority had been tested on their first visit to CHIEDZA (*n* = 28) with the remainder (*n* = 3) being tested on their second visit. Twenty‐nine participants chose provider‐initiated testing (FGD *n* = 21, IDI *n* = 4 and paired interviews *n* = 4), and two opted to conduct self‐testing (IDI *n* = 2) in the on‐site booth. Those who took a kit for off‐site testing could not be included as contact details were only for clinical follow‐up and not for research purposes. Interviews were conducted at the CHIEDZA sites between 1 and 7 weeks after their test.

A trained qualitative researcher (CM) conducted the FGDs and IDIs in Shona, using method‐specific topic guides. All FGDs and IDIs were audio‐recorded, with two IDI exceptions where recording was refused due to concerns about confidentiality and detailed notes were taken instead. All recordings were transcribed in English. Each interview lasted between 25and 50 min and FGDs between 40and 60 min.

Iterative thematic analysis was used to explore both deductive themes identified before data collection and inductive themes, which emerged from the data [18]. NVivo 12, a qualitative data management and analysis software, was used to aid coding and analysis [19]. Data collection continued until thematic saturation was reached (i.e. new data had become broadly repetitive of previously collected data in regards to the key themes) [20].

### 2.4 Ethical considerations

Ethical approval was granted by the Medical Research Council of Zimbabwe, the Biomedical Research and Training Institute Institutional Review Board and the London School of Hygiene and Tropical Medicine ethics committee. Written informed consent was obtained from all clients interviewed. A waiver for the requirement of guardian consent was granted for 16‐ and 17‐year‐olds (24/31 participants).

## 3 RESULTS

### 3.1 Uptake of HIV testing

Between 1 April and 27 June 2019, a total of 1924 clients attended CHIEDZA centres for the first time. There were 1476 females (76.61%), and 979 (50.88%) were aged between 20 and 24 years, with median age (IQR) of 19 [17, 18, 19, 20, 21, 22]. In total, 1415 (73.5%) clients were eligible for HIV testing; 879 (62.1%) were first time testers (i.e. had an unknown status). Of all the clients who accepted testing, the proportion tested among those who had tested negative before (repeat testers) was higher than the proportion tested among those who had unknown status (first time testers) (73.32% vs. 63.48 *p*<0.001). Of all clients eligible for testing, 1097 (77.53%) were eligible at their first visit, 225 (15.90%) were eligible at the second visit (clients who did not take testing up at the first visit and still had unknown HIV status or 6 months between HIV tests had elapsed by the time of the second visit). Only 93 (6.57%) were eligible beyond their second visit.

Overall, 951 (67.2%) accepted HIV testing. Provider testing accounted for 898 (94.4%) of HIV tests done. Private self‐testing in a booth accounted for 30 (3.2%) and off‐site HIVST accounted for 23 (2.4%) of the HIV testing conducted (Table 1). Of the 951 clients who accepted testing, 732 (76.97%) were tested at their first visit, 152 (15.98%) at the second visit and 67 (7.05%) in subsequent visits. Only 251 (26.39%) were males and 49.63% were aged 16‐ to 19‐year‐olds. The HIV prevalence among those who tested was 1.05% (10/951) (Table 1).

**Table 1:** Proportion of eligible clients tested using three different testing strategies

			Testing mode		
		All	Provider testing	HIVST at site	Off‐site HIVST
All	*N*	951	898	30	23
Female	*n*	700	665	20	15
	%	73.61	74.05	66.67	65.22
16–19 years	*n*	472	452	17	3
	%	49.63	50.33	56.67	13.04
Cluster 1	*n*	293	272	10	11
	%	30.81	30.29	33.33	47.83
Cluster 2	*n*	261	256	2	3
	%	27.44	28.51	6.67	13.04
Cluster 3	*n*	187	175	9	3
	%	19.66	19.49	30.00	13.04
Cluster 4	*n*	210	195	9	6
	%	22.08	21.71	30.00	47.83
Reactive	*n*	10	10	0	0
	%	1.05	1.11	0.00	0.00
Non‐reactive	*n*	929	888	30	11
	%	97.58	98.89	100.00	47.83
Indeterminate	*n*	1	0	0	1
	%	0.11	0.00	0.00	4.39
Lost to follow‐up (test result not entered)	*n*	11	0	0	11
	%	1.16	0.00	0.00	47.83

### 3.2 HIV testing preferences

Reasons for clients’ selection of particular models of HIV testing were explored qualitatively. All participants were asked about their most recent HIV test. For 28 out of the 31 participants in the qualitative study, the HIV test done at the CHIEDZA site was their first time being tested. A key characteristic noted across this group was that although they appeared comfortable to elaborate certain topics, such as why they attended CHIEDZA, the majority of participants, most notably in the IDIs, tended to talk in relatively concise statements about their HIV testing experiences. This may indicate a lack of familiarity and confidence in talking about this topic. The data presented are anonymized and contextual explanation is provided for a number of the briefer extracts. Table 2 details supplementary quotes, which reflect the pertinence of the themes across the dataset.

**Table 2:** Supplementary quotes to support the qualitative themes in the study

Themes	Quotes to support them
Confidence in the youth‐friendly environment	‘*This is good hospitality offered here at CHIEDZA, different from what is done in clinics and hospitals by the nurses who don't care about patients but focus on doing what makes them get paid at the end of the month…*’ (FGD2, 17‐year‐old female, provider tested)
Barriers to HIVST
*Lack of confidence in their expertise in using the oral test‐kit*	Most participants ‘*had never heard about*’ self‐testing (paired IDI1, 17‐year‐old female, provider tested) ‘*Personally, for me I had never heard about it. When I came to Chiedza, that's when I knew that there is an HIV test kit which is called self‐testing and I enjoyed the experience…*., *“the person [provider] fully explained everything such that I understood what they were saying*”’ (paired IDI1, 17‐year‐old female, provider tested).
*Fear of finding out test results alone*	Many young people discussed fears of ‘*the results coming out as HIV positive*’ leading to ‘*mental problems*’ and even ‘*you might think of killing yourself*’ (FGD2 and FDG4, both 16‐year‐old females, provider tested). ‘*Proper support system to comfort me when I needed the support*’ at home was the key aspect (paired interview 2, female, 24 years old, provider tested)

Overall, there were no gendered patterns in the preferences and experiences of HIV testing. The dominant explanation for the strong preference for provider testing at CHIEDZA was that it was perceived to be the highest quality option. Participants identified three key indicators of quality.

#### 3.2.1 Confidence in the youth‐friendly environment

The young participants trusted CHIEDZA providers. Despite being anxious about their results, participants expected that the CHIEDZA testing process would be youth‐friendly, non‐judgemental and confidential. Some participants even came to CHIEDZA for the first time with the specific intention of getting tested, encouraged by the positive reviews from their peers.

‘…since it [HIV testing] is done here at CHIEDZA, the staff treats you well. They don't scold you, even though they are meeting you for the first time. This is the good hospitality offered here at CHIEDZA’ (FGD2, 18 years, female, provider tested)

This was in stark contrast to their negative experiences at the other health facilities. The approachability and confidentiality of staff at CHIEDZA were described as being significant incentives to test.

‘At hospitals, the staff are very harsh and rough, such that you may sit there at the reception for quite some time with no‐one attending to your needs. And this is different from here at CHIEDZA, when you enter the hall the staff smiles at you to make you feel welcome hence making the conversations that we have very worthwhile’ (paired IDI2, 19 years, female, provider tested)

#### 3.2.2 Trust in providers’ expertise

Participants reported having supported discussions with providers around oral HIV testing. While oral HIV testing was novel to all study participants, they described being confident in the expert test administration and efficiency of the providers.

‘I was surprised being told (at CHIEDZA) that we have an easier way of testing for HIV…The community health worker showed me how it's done…Whilst we are busy talking about other issues, the process will be happening and after some minutes the results will be ready and it's easy… ’ (FGD2, 17 years, female, provider tested)

#### 3.2.3 Face‐to‐face counselling mitigated anxiety and provided accessible support

Young people derived considerable value from the pre‐and post‐test counselling from the health provider as it meant that they had immediate access to ‘*support throughout*’ (FDG4, 16 years, female, provider tested) the testing experience. Having the comforting presence of the provider made the immediate waiting time more bearable, assauged their anxiety and made the experience lighter. They credited the providers’ reassuring support as being a critical component in being able ‘*to accept our own results*’, and to help avoid ‘*living [my] life in denial*’ (IDI5, 23 years, female, provider tested). This enabled them to approach testing with more confidence that should they test positive that they would link into care and intiate treatment.

A young woman explained her preference for provider testing over the self‐testing options, reflecting a rationale which was widely shared among participants.

‘I personally prefer when there is someone present because as youths we have a tendency of refusing to accept our own results. Let's say I had used the self‐test and the results came back positive I would start to live my life in denial. So if a health professional is closer, you will receive counselling to receive whatever results that would have come out. So I personally prefer getting tested with a health professional nearby, maybe I may test myself using the self‐test kit but a health professional must be around so that when the results come out I will get the appropriate counselling to move on and accept the result’ (IDI7, female, 18 years old, provider tested)

Young people also considered that provider‐delivered testing potentially amplified their access to familial support should they receive a positive result. Participants emphasized how the circumstances of condomless sex, which may have prompted them to take the HIV test, would not be an experience that they could discuss with their parents. A young woman explained that had she found out that she was positive after self‐testing she would feel alone in the struggle to tell her family, which would impede her ability to gain their support. In contrast, provider testing meant that she would ‘*be able to receive the proper counselling and support with the professional counsellor being able to tell parents, with consent of course, that this is what has transpired hence having a stronger support system*’ (FGD5, 16‐year old, female, provider tested).

### 3.3 Barriers to HIVST

The significant preference for provider testing was also influenced by four key barriers that discouraged HIVST.

#### 3.3.1 Lack of confidence in their expertise in using the oral test‐kit

The novelty of HIVST meant that many participants lacked the confidence to administer the test and interpret the result themselves. Several young people had feared ‘*failing to follow the instructions*’ properly at home (IDI6, 17 years, female, provider tested) and were worried that this could lead to a ‘*false positive*’ (IDI5, 23 years, female, provider tested). A young woman explained that self‐testing might appeal to someone who did not trust their local clinic, but given that she did trust the providers and services at CHIEDZA, the risks of self‐testing had been too great to entertain. She emphasized the importance of having someone with expertise present:

‘If it's your first time getting tested obviously you are going to make some mistakes and need some tips on how to do it. To make sure you get a good result you have to have someone next to you who understands more about the kit and HIV testing’ (IDI5, female, 23, provider tested)

#### 3.3.2 Lack of privacy

Young people described having limited autonomy to ensure privacy at home and considered that the CHIEDZA sites facilitated better access to private spaces to conduct the test. A young man noted that testing at home, without a private space, would have also made the process of checking his HIV status ‘*more challenging and stressful*’ (IDI2, 19 years, male, self‐tested on site).

#### 3.3.3 On‐site self‐testing feels like it takes longer

A very small minority of clients chose to self‐test in a private booth within CHIEDZA *n* = 30 (3.15%) (Table 1). This small group valued both the privacy and autonomy of self‐testing, alongside the reassurance of support and being within the youth‐friendly provider system at CHIEDZA. However, there was one private booth for self‐testing at CHIEDZA and if it was already occupied, there could be long waiting times. Providers considered that this may have influenced uptake as many clients would either *‘end up leaving’* without getting tested or *‘will opt for the provider one’* (FGD3, CHIEDZA health provider).

Waiting times were not cited as a barrier by young people though. Rather, they described how testing on your own would make the wait feel longer and even more ‘*stressful*’ (FGD2, 16‐year‐old female, provider tested). Testing in the company of the provider, with whom they had an existing rapport and felt safe with, made the time spent waiting for the result more bearable.

#### 3.3.4 Fear of finding out test results alone

This relates to the final key barrier: they did not want to find out that they had an HIV‐positive status on their own. Many clients highlighted the challenges this would pose for their mental health as an HIV diagnosis is still considered ‘*a death sentence*’ by some and several clients specifically mentioned that they ‘*would commit suicide after seeing the results*’ alone at home (FGD2, 17 years, female, provider tested). This exacerbated their anxiety about the testing process.

‘Taking the test home was not as option because one might lie to oneself about the positive test result when there was no ready support available’ (IDI1, 20 years, female, self‐tested on site).

The providers noted that as most clients were testing for HIV for the first time (62.1%), these new testers needed guidance and support. They suggested that HIVST was not appropriate for this age group, unless they had already experienced being tested many times before.

‘I personally think that yes the provider assisted is the best for the 16–24 ages. Even if they come they will tell you that I'm worried as they wait for the results, which simply confirms the reason why they have never tested all this time. Self‐testing is something that is good but I think let's appreciate the ages that we are dealing with’ (FDG3, CHIEDZA health provider).

Given the timeframe of the study, youth tested for the first time at CHIEDZA had not yet reached the point of being repeat testers, and this emerging hypothesis could not be tested with the current data.

## 4 DISCUSSION

We conducted a comparative analysis of the uptake and acceptability of provider‐facilitated testing and two HIVST options. The young people in the study overwhelmingly preferred provider testing because it was conducted at a trusted youth‐friendly service within the community by expert staff, whom they anticipated would provide effective in‐person support throughout the process. Barriers to HIVST for youth included the fear of testing by themselves without support and counselling, the lack of privacy at home, limited confidence in their ability to conduct a self‐test and accurately interpret the results and concerns around being able to cope with a positive result. Without conducive conditions in place for HIVST, which were identified as trust in their expertise and access to private spaces, and if they had access to youth‐friendly provider testing, then HIVST for youth comparatively exacerbated rather than ameliorated youth's hesitancy to engage in HIV testing.

CHIEDZA was intentionally introduced to serve youth who are averse to health facilities, and was established as a youth‐friendly space, which includes counselling, social activities during wait times, non‐judgemental providers, and assures clients of privacy and confidentiality. The preference for provider testing appears to be influenced by the provision of this quality service. These findings support literature that demonstrates the value of quality provider care during the HIV testing process for both adults and youth [21, 22, 23]. The need for support is not exclusive to young people. In Uganda, among groups with elevated HIV exposure, such as adult fishermen and sex workers, the absence of a health professional and poor linkage to care provoked hesitancy about HIVST [24].

Despite the community setting, integrated service provision, and choice of options, getting youth to test remains challenging. About one‐third of eligible young people chose not to test within the short timeframe of this study. However, among those who did test, our study counters an increasingly dominant narrative that young people primarily desire autonomy and privacy in health service engagement [25], and instead demonstrates that where provider testing is non‐judgemental and youth‐friendly, this is the preferred option.

The CHIEDZA providers perceived that first time testers would be more averse to HIVST compared to repeat testers. However, testing uptake in CHIEDZA showed that even among repeat testers, as well as first‐time testers, within the context of a youth‐friendly, intervention provider testing was preferred. This demonstrates that these provider‐related aspects of quality care are hugely valued by young people. The pathway to improving uptake of HIV testing among this demographic may be through increasing investment in the provision of services which are underpinned by an ethos of acceptance and support. Analyses of the trial findings of the intervention (run over 24 months) will provide clearer evidence, compared to the 11‐week timeframe adopted for this study.

There is an increasing body of research, which, in seeking to understand decision‐making in HIV testing, emphasizes that the act of testing is not experienced as a singular event, but is inherently linked to the full HIV continuum, from initially recognizing HIV risk to being able to engage in life‐long HIV treatment and care [10, 26, 27, 28, 29, 30]. Individuals’ decision‐making processes of when, how and where to test need to be understood within this broader biographical, social and clinical context. In line with recent studies conducted in Zimbabwe [29, 30], the young people in this study emphasized the positive effects of being able to exercise agency and autonomy by engaging in testing at a time of their choosing and in spaces where they felt supported. Their preference was to have a trusted, professionally trained individual with them to both administer the test and also to support them in managing the implications of a positive result, including engagement in treatment and potentially facilitating a supportive response from their families.

These study findings caution against the interpretation of the desire for autonomy as being synonymous with self‐administered services, which can be experienced as disempowering. Young people did not feel that they had the requisite expertise or capacity to accurately administer the test and respond to the results on their own. Although young people emphasized that discretion is desired in HIV testing, they were simultaneously emphatic that this did not necessarily equate to undertaking the test on their own. To design and deliver youth‐tailored HIV and SRH interventions more broadly, it is vital to identify with young people in what ways they want to exercise agency to feel empowered to engage in services, so that we can attend to the nuance of what is meant by a desire for autonomy.

Variation in the acceptability and uptake of HIVST may reflect the profile of local service provision or standard of care services. HIVST may be more appealing when quality provider‐facilitated youth services are absent. Considering the CHIEDZA context, where off‐site HIVST package was linked to a smartphone App, it is possible that self‐testing may increase as access to smart phones with the relevant technologies continues to increase across this age group. Additionally, high uptake of HIVST has been established among key risk groups, such as MSM, sex workers and pregnant women [6, 12], who are often repeat testers. They may not need continued guidance and support when compared to young people who often may be testing for the first time. In Zimbabwe and Malawi, when HIVST was compared to services at health facilities, young people valued the privacy and autonomy of community home distribution of HIVST, where the alternative was provider testing in a clinic where they lacked confidence in the confidentiality and quality of support and counselling [25]. HIVST may, therefore, present as a suitable option for young people who have access to their own private spaces and have limited testing alternatives. However, our findings show that unless there is private space, knowledge of HIV and linkage to care options, the appeal of HIVST is diluted for young people. HIVST is an option to be used alongside, rather than instead of, investment in improving quality of service provision.

During recruitment to participate in the study, the majority of the young people preferred to participate in group (FGD and paired depth interview) settings suggesting the value of relational support in engaging in the testing process. The accounts given in the individual interviews tended to be more succinct than those elicited through group interviews. Experiencing the group interview process with peers may have created a more ‘socially safe’ space to talk about their HIV testing experiences. The influence of being in trusted company to increase young people's confidence to engage in discussions about HIV testing appears to align with the logic underpinning their preference for provider testing. For young people, what constitutes a ‘safe’ HIV care journey may be more orientated towards experiencing testing with people who they trust, rather than seeking out the solitude offered by HIVST, especially when it may threaten rather than secure privacy. The inclusion of assistance, support and counselling when conducting HIV tests remains paramount for young people.

The study had several limitations. Participants in this study were clients who had come to a CHEDZA site. We were unable to capture the experiences of youth within the community who were eligible but not attending CHIEDZA. Given the novelty of some participants’ experience of testing and talking about testing, some participants provided brief, concise statements in their interviews. This may have been further influenced by the interview dynamics, as young people appeared more comfortable talking in group settings. The off‐site HIVST option was exclusive to youth with a smartphone and the experiences of these clients were not captured. We did not track the youth who were excluded from off‐site HIVST due to smartphone technology constraints. The trial had very low numbers of those who self‐tested, and they could not be readily accessed once they had taken the HIVST kit off site. Further research with youth who self‐tested and/or with youth for whom self‐testing is the only HIV testing option is required to understand what underpins the appeal of this option, as well as the potential impact of limited access to appropriate technology to support uptake. This also includes research on how HIVST can be set up for youth who are not engaging with health services.

## 5 CONCLUSIONS

For young people, accessing trusted counselling and support is vital to encourage the uptake of HIV testing. Our findings suggest the primary need for investment in providing supportive, non‐judgemental and effective provider testing, as this may be the preferred and optimum route for HIV testing among young people. Although HIVST is appealing to various adult groups, our study demonstrates that HIVST may have limited pertinence for young people who do not have access to private spaces outside of the clinic and fear finding out about an HIV‐positive diagnosis alone and unsupported. HIVST may be an option in certain conditions, but it should not detract from investments in improving quality provider care.

## COMPETING INTERESTS

None declared.

## Authors’ contributions

CM and RAF conceptualized the study. CM collected and analysed the qualitative data with support from CMY and SB, and wrote the first draft. TB analysed the quantitative data. ED, CDC, MT, CM, KK and RAF implemented the CHIEDZA trial. RAF is the PI of the CHIEDZA trial.
